# Sporadic Occurrence of Enteroaggregative Shiga Toxin–Producing *Escherichia coli* O104:H4 Similar to 2011 Outbreak Strain

**DOI:** 10.3201/eid2809.220037

**Published:** 2022-09

**Authors:** Claudia E. Coipan, Ingrid H. Friesema, Maaike J.C. van den Beld, Thijs Bosch, Sabine Schlager, Menno van der Voort, Christina Frank, Christina Lang, Angelika Fruth, Eelco Franz

**Affiliations:** National Institute for Public Health and the Environment (RIVM), Bilthoven, the Netherlands (C.E. Coipan, I.H. Friesema, M.J.C. van den Beld, T. Bosch, E. Franz);; Institute for Medical Microbiology and Hygiene, Graz, Austria (S. Schlager);; Wageningen Food Safety Research, Wageningen, the Netherlands (M. van der Voort);; Robert Koch Institute, Berlin, Germany (C. Frank);; Robert Koch Institute, Wernigerode, Germany (C. Lang, A. Fruth)

**Keywords:** Shiga toxin-producing Escherichia coli, STEC, zoonoses, foodborne infections, public health, enteroaggregative E. coli, O104:H4, bacteria, enteric infections, food safety, the Netherlands, Austria

## Abstract

We describe the recent detection of 3 Shiga toxin–producing enteroaggregative *Escherichia coli* O104:H4 isolates from patients and 1 from pork in the Netherlands that were genetically highly similar to isolates from the 2011 large-scale outbreak in Europe. Our findings stress the importance of safeguarding food supply production chains to prevent future outbreaks.

Shiga toxin–producing *Escherichia coli* (STEC) is a zoonotic pathogen that causes illness ranging from mild diarrhea to hemolytic uremic syndrome and death. During 2011, an exceptionally large outbreak caused by serotype O104:H4 STEC occurred in Europe, mainly in Germany and France, that was associated with sprouts grown from imported fenugreek seeds (*1*). Besides the ability to produce Shiga toxin, specifically *stx2a*, the strain had the genetic characteristics and phylogenetic backbone of an enteroaggregative *E. coli* (EAEC) pathotype (*2*) but lacked other classical STEC virulence markers *eae* and *hlyA* (*3*). In addition, the outbreak strain carried plasmid-borne *bla*_CTX-M-15_ and *bla*_TEM-1_ genes. The epidemiologic investigation revealed that a contaminated batch of fenugreek seeds imported into the European Union from Egypt was the most probable source of the pathogen causing the outbreak (*4*). 

After the 2011 outbreak in Germany and France, only a few sporadic cases of infection with Shiga toxin–producing EAEC O104:H4 were reported, most related to travel to Turkey or North Africa (*5*–*8*). We describe the sporadic occurrence of Shiga toxin–producing EAEC O104:H4 isolates in the Netherlands, originating from 2 clinical cases from 2019 and 2020 and 1 food isolate from 2017. In addition, we report a clinical case from Austria in 2021.

## The Study

Surveillance of Shiga toxin–producing *E. coli* (STEC) in the Netherlands is performed in 1 or both of 2 ways: by mandatory case notification from medical laboratories or physicians to regional public health services, which then report the cases to the Netherlands National Institute for Public Health and the Environment (RIVM), or by microbiological surveillance, when medical laboratories send suspected STEC isolates to RIVM for confirmation and further molecular characterization. Since 2016, STEC isolates have been subjected to whole-genome sequencing for high-resolution detection of clusters and outbreaks nationwide. During detailed retrospective analysis of the whole-genome sequencing data, we identified isolates from 2 clinical cases of STEC infection from 2019 and 2020 and characterized them as *stx2a*-encoding EAEC O104:H4 (isolates NL1981 and NL2076). Both cases involved middle-aged nonhospitalized women with abdominal cramps and bloody diarrhea. Neither of the 2 case-patients reported recent travel. The 2019 case-patient reported consuming cooked minced beef, and the 2020 case-patient reported consuming beef, hamburger meat, and vegetables and fruits from her own garden. In 2021, the Austrian Agency for Health and Food Safety obtained a sample of similar *stx2a*-encoding EAEC O104:H4 from a 10-year-old girl with hemolytic uremic syndrome. This case-patient also reported no recent travel, but she had consumed raw veal. Finally, we retrospectively identified *stx2a*-encoding EAEC O104:H4 in a sample obtained from pig meat (pork) in 2017 in the Netherlands.

**Figure 1:**
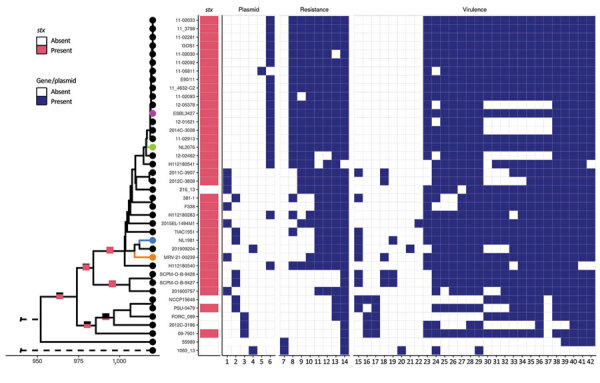
Single-linkage hierarchical clustering tree of enteroaggregative Shiga toxin–producing *Escherichia coli* O104:H4 from the Netherlands and reference sequences. Tree results from core- and accessory-genome multilocus sequence typing with a heatmap indicating presence or absence of *stx*-encoding bacteriophage, plasmids, resistance, and virulence genes. Only genes present in at least 1 isolate are depicted. Colored isolates are those added during this study: green indicates the patient isolate from the Netherlands in 2020, purple the pork isolate from the Netherlands in 2017, blue the patient isolate from the Netherlands in 2019, orange the patient isolate from Austria in 2021. The stacked bar plots on a few selected branches in the tree indicate the likelihood at the downstream nodes of having contained an *stx*-encoding phage (black: absence, pink: presence). Lane 1, IncFII(prSB107); lane 2, IncB/O/K/Z; lane 3, IncFIB(AP001918); lane 4, IncFII; lane 5, Col(BS512); lane 6, Incl1-l(Alpha); lane 7, *tet(B)*; lane 8, *bla*_CTM-M-15_; 9, *tet(A)*; lane 10, *aph*(3′′)-lb, *aph*(6)-id, *sul2*; lane 11, *dfrA7*; lane 12, *qacE*, *sul1*; lane 13, *bla*_TEM-1B_; lane 14, *formA*, mdf(A); lane 15, *traT*; lane 16, *agg3A*, *agg3D*; lane 17, *agg3B*, *agg3C*, *astA*; lane 18, *iss*; lane 19, *celb*; lane 20, *aadA1*, *aadA2b*, *ant*(3′′)-la *bla*_OXA-1_, *catA1*, *eatA*; lane 21, *ireA*; lane 22, *hra*; lane 23, *neuC*; lane 24, *gad*; lane 25, *mchB*; lane 26, *mchC*; lane 27, *stx2A*, *stx2B*; lane 28, *aaiC*, *capU*, *iucC*, *lpfA*; lane 29, *terC*; lane 30, *aatA*; lane 31, ORF3; lane 32, ORF4; lane 33, *aap*; lane 34, *aggR*; lane 35, *aar*; lane 36, *afaD*; lane 37, *aggA*, *aggB*, *aggC*, *aggD*, *sepA*; lane 38, *mchF*; lane 39, *iha*; lane 40, *iutA*, *pic*, *sigA*; lane 41, *fyuA*; lane 42, *irp2*. ESBL, extended spectrum β-lactamase; ORF, open reading frame.

**Figure 2:**
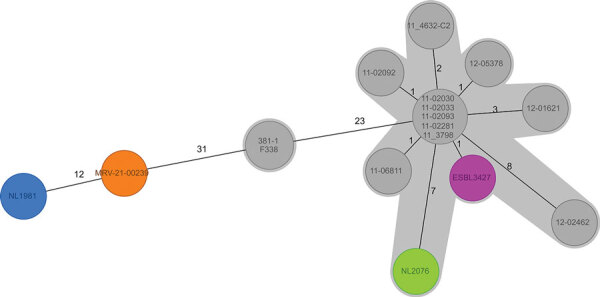
Minimum-spanning tree from cgMLST (Enterobase STEC scheme) of enteroaggregative Shiga toxin–producing *Escherichia coli* O104:H4 from the Netherlands and reference sequences. Colored isolates are those added during this study: green indicates the patient isolate from the Netherlands in 2020, purple the pork isolate from the Netherlands in 2017, blue the patient isolate from the Netherlands in 2019, orange the patient isolate from Austria in 2021. The gray-shaded area connects strains that have an allelic distance <10. ESBL, extended spectrum β-lactamase.

We performed a genomic comparison among the recently identified isolates, 2 Shiga toxin–producing EAEC O104:H4 isolates in cases from the Netherlands from 2013 described elsewhere (*8*), and a set of representative isolates from a 2011 outbreak in Germany and France, including isolates from case-patients in Germany who shed the outbreak strain well into 2012 (long-term shedders). To achieve our full set of isolates (Table), we supplemented the dataset with O104:H4 isolates retrieved from public repositories to gain more insights into the relatedness among investigated isolates and track possible evolutionary events. We assessed genomic relations between isolates by core- and accessory-genome multilocus sequence typing using the Enterobase STEC schema implemented in Ridom Seqsphere version 8.0.1 (https://www.ridom.de). The resulting dendrogram was rooted with strain 1060_13, a Clermont type A isolate. The 2020 patient isolate from the Netherlands (NL2076) and the pork isolate (ESBL3427) clearly clustered with the representative outbreak isolates (Figures 1,2). Conversely, patient isolates NL1981 from the Netherlands in 2019 and MRV-21-00239 from Austria in 2021 appeared more similar to each other and the 2 patient isolates (338 and 381_1) from the Netherlands in 2013 than to isolates from the outbreak cluster (Figures 1,2).

**Table:** Overview of strains used in study of enteroaggregative Shiga toxin–producing *Escherichia coli* O104:H4 from the Netherlands*

Strain	Country	Source	Year	Reference/source
11-4632-C2	France	Patient	2011	(*13*)
11-3798	Germany	Patient	2011	(*13*)
11-02030	Germany	Patient	2011	(*13*)
11-02033	Germany	Patient	2011	(*13*)
11-02092	Germany	Patient	2011	(*13*)
11-02093	Germany	Patient	2011	(*13*)
11-02281	Germany	Patient	2011	(*13*)
11-06811	Germany	Patient	2011	(*14*)
F338	Netherlands	Patient	2013	(*8*)
381-1	Netherlands	Patient	2013	(*8*)
12-01621	Germany	Long-term shedder	2012	†
12-02462	Germany	Long-term shedder	2012	†
12-05378	Germany	Long-term shedder	2012	†
ESBL3427	Netherlands	Pork	2017	†
NL1981	Netherlands	Patient	2019	†
NL2076	Netherlands	Patient	2020	†
MRV-21-00239	Austria	Patient	2021	†
2015EL-1494M1	United States	Human	NA	Enterobase
TIAC1951	Belgium	NA	2003	Enterobase
09-7901	France	NA	2009	Enterobase
2012C-3196	United States	NA	2012	Enterobase
FORC_069	South Korea	Human	2016	Enterobase
PSU-0479	South Korea	NA	2000	Enterobase
NCCP15648	South Korea	Human	2001	Enterobase
1060_13	United Kingdom	Human	2014	Enterobase
55989	Central African Republic	Human	2002	Enterobase
201909204	France	Human	2019	Enterobase
216_13	United Kingdom	Human	2014	Enterobase
SCPM-O-B-9428	Russia	Human	2018	Enterobase
SCPM-O-B-9427	Russia	Human	2018	Enterobase
201600757	France	Human	2016	Enterobase
2011C-3907	United States	NA	2011	Enterobase
2012C-3808	United States	Human	2012	Enterobase
2014C-3008	United States	NA	2013	Enterobase
E90/11	Germany	Human	2011	Enterobase
GOS1	NA	Human	2011	NCBI
H112180541	United Kingdom	Human	2011	NCBI
H112180283	United Kingdom	Human	NA	NCBI
H112180540	United Kingdom	Human	NA	NCBI
11-02913	Germany	Human	2011	Enterobase

*NA, not available; NCBI, National Center for Biotechnology Information. †Genome sequence raw data files are available from the European Nucleotide Archive (projects PRJNA174138 and PRJEB49642).

We determined the content of the plasmid, virulence, and antimicrobial resistance genes using the PlasmidFinder, VirulenceFinder, and ResFinder databases (http://www.genomicepidemiology.org). Within the outbreak cluster, including the patient (NL2076) and pork (ESBL3427) strains from the Netherlands, isolates showed virulence profiles typical of the 2011 outbreak strain, including STEC virulence markers *stx2a, lpfA,* and *iha*; the EAEC pAA plasmid (with *aggR*, *aar, aap*, *sepA*, the *aatPABCD* operon, and the *aggABCD* operon); and some chromosomal EAEC markers such as *pic* and *sigA*, but they lacked the STEC hallmark intimin *eae*. In addition, the isolates within the outbreak cluster showed similar antimicrobial resistance profiles, including *bla*_CTX-M-15_ and *bla*_TEM-1B_. Exceptions were the 2012 isolates from long-term shedders in Germany, which lost the pAA plasmid but belong to the outbreak cluster on the basis of their core genome (*9*) (Figure 1). Ancestral trait reconstruction as implemented in the R package ape v.5.5 (*10*), indicated a high likelihood that the most recent common ancestor of the isolates identified in this study were lysogenized with an *stx*-encoding phage. Among the isolates in the outbreak cluster, the core-genome allelic distance was 0–15 alleles (Figure 2).

## Conclusions

After the 2011 outbreak in Europe, only a few sporadic cases of infection with *stx*-producing EAEC O104:H4 strains, most related to travel to Turkey or North Africa (*8*,*11*), were reported. The fact that the particular EAEC O104:H4 strains have never convincingly been isolated from food, animal, or environmental sources, and that EAEC in general are primarily human-adapted (*12*), support the hypothesis of a human reservoir and potential multiple events of import by travelers from an area where this pathogen is endemic. On the other hand, the loss of essential virulence markers in long-term shedders might be a preview of common trends of bacterial genome reduction in long-term carriage in humans which, in turn, might indicate that the newly signaled isolates are maintained in niches other than the human one. Genomic analysis of several post-outbreak EAEC O104:H4 isolates suggests that they are not derived from the 2011 outbreak but share a recent common ancestor (*13*). Our analysis indicates that distinct more- or less-distant variants of Shiga toxin–producing EAEC O104:H4 are circulating worldwide. These variants are more likely to represent independent evolutionary events than continuous diversification of a single clade established and circulating in Europe after the large 2011 outbreak (*14*). In the absence of any previous indication of Shiga toxin–producing EAEC O104:H4 in animals, it was surprising to retrieve such an isolate from a pork product. However, this finding does not necessarily imply that pigs are a reservoir, because the contamination could also have originated from a food handler or contaminated feed rather than the pigs.

In conclusion, we show that Shiga toxin–producing EAEC O104:H4 isolates highly related to the 2011 outbreak strain are sporadically occurring in Europe. We emphasize the need to optimize safeguarding vulnerable chains of food production to prevent future outbreaks.
